# AI-Driven Smart Cockpit: Monitoring of Sudden Illnesses, Health Risk Intervention, and Future Prospects

**DOI:** 10.3390/s26010146

**Published:** 2025-12-25

**Authors:** Donghai Ye, Kehan Liu, Chenfei Luo, Ning Hu

**Affiliations:** 1 School of Arts, Chongqing University, Chongqing 400044, China; yedonghai@cqu.edu.cn (D.Y.);; 2 School of Bioengineering, Chongqing University, Chongqing 400044, China; 3 School of Automation, Chongqing University, Chongqing 400044, China

**Keywords:** disease surveillance, intelligent vehicle cockpit, risk intervention

## Abstract

Intelligent driving cabins operated by artificial intelligence technology are evolving into the third living space. They aim to integrate perception, analysis, decision making, and intervention. By using multimodal biosignal acquisition technologies (flexible sensors and non-contact sensing), it is possible to monitor the physiological indicators of heart rate and blood pressure in real time. Leveraging the benefits of domain controllers in the vehicle and edge computing helps the AI platform reduce data latency and enhance real-time processing capabilities, as well as integrate the cabin’s internal and external data through machine learning. Its aim is to build tailored health baselines and high-precision risk prediction models (e.g., CNN, LSTM). This system can initiate multi-level interventions such as adjustments to the environment, health recommendations, and ADAS-assisted emergency parking with telemedicine help. Current issues consist of sensor precision, AI model interpretation, security of data privacy, and whom to attribute legal liability to. Future development will mainly focus on cognitive digital twin construction, L4/L5 autonomous driving integration, new biomedical sensor applications, and smart city medical ecosystems.

## 1. Introduction

With the rapid development of automobile-related technologies, the smart cockpit, which is an essential part of intelligent and connected vehicles, has gradually transformed from a simple means of transportation into a “third living space”. The comfort level, intelligence level, and human–machine interaction experience of smart cockpits have increasingly attracted attention [1,2,3,4]. Smart cockpit health monitoring and risk intervention can essentially improve driving safety, enhance passenger comfort, and meet the demand for in-vehicle health management [5].

**Figure 1:**
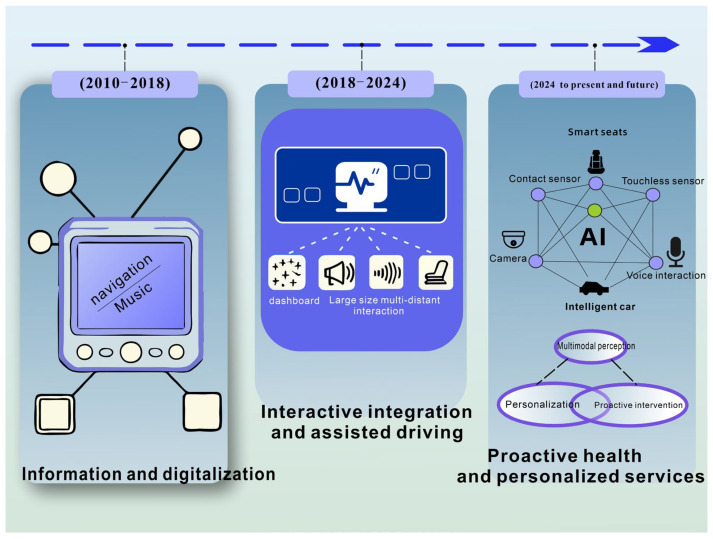
The evolution of informatization and intelligentization in automotive smart cabins.

Early cockpit designs focused on driving assistance functions and mass flow meters, but not overall driving capability. For instance, the idea of an AI-supported cockpit proposed in 1985 examined the possibility of using artificial intelligence on all sorts of assistive systems in commercial transport [6]. Today, the smart cockpit is no longer an infotainment system, but a complex platform with advanced driver assistance systems (ADASs) and human–machine interaction (HMI) thanks to the rapid development of Internet of Things (IoT) and vehicle computing technologies [7,8]. As one of the most important future platforms for human–computer interaction (HCI) and human–machine interfaces (HMIs), the smart cockpit has also become a major application scenario for large language models (LLMs) [9]. As illustrated in Figure 1, during 2010–2018, cockpit design centered around the central control screen, which provided basic functions such as navigation and entertainment. Between 2018 and 2024, through the deep integration of multi-screen systems and ADASs, driver monitoring systems (DMSs) were introduced to track driver states—though their intervention capability remained limited. Since 2024, smart cockpits have adopted contact and non-contact sensors to continuously collect physiological data, build personalized models, assess health risks, and trigger individualized interventions, thereby forming a complete “monitoring–assessment–intervention” closed loop.

Currently, the rapid advancement of biosensor technologies has laid a solid foundation for integrating health monitoring systems into automotive technologies. Meanwhile, research on AI-based management and control within smart cockpits is accelerating the intelligent transformation of automobiles. Techniques such as deep learning, reinforcement learning, and deep reinforcement learning have become the core of domain controllers, providing essential technical support for achieving advanced levels of health monitoring and intervention in smart cockpits [10].

### 1.1. Integration Trends of Artificial Intelligence and Smart Cockpits

The rapid advancement of artificial intelligence (AI) is driving a major transformation in automotive smart cockpits, where the application of AI has undergone comprehensive upgrades at the levels of chips, sensing technologies, and algorithms [11,12]. In smart cockpits, AI enables more natural and intelligent human–vehicle interaction through technologies such as voice recognition, natural language processing (NLP), and multi-turn dialog systems. For example, recent studies have proposed AI-based voice interaction systems for smart cockpits, which leverage high-precision multimodal perception architectures, edge computing deployment strategies, and vehicle–road collaborative ecosystems to support online monitoring and driver safety management [13]. In addition, AI is critical for vehicle perception and driving decision making. Systems can actively perceive drivers’ emotions, which is vital for road safety [14].

### 1.2. Challenges in Driver Health and Safety

A lot of traffic accidents occur due to the physical and mental condition of drivers. According to official statistics, the human factor is among the main causes of road traffic accidents [15]. For example, distractions, driving while tired, driving while drunk or under the influence of drugs, and emotional instability can increase accident risk. Specifically, long-distance driving and complex traffic conditions require drivers to maintain a high level of concentration for a long time. Such situations can cause both physical and psychological fatigue, resulting in poor judgment and reaction time. As shown in Table 1, when an acute health incident occurs, e.g., due to cardiovascular diseases or epileptic seizures, while driving, the absence of timely monitoring or intervention mechanisms may lead to serious situations [16,17]. The primary focus of traditional driver assistance systems (DASs) is physical safety at the vehicle level, such as avoiding a collision or alerting the driver if they drift off course. The functionality of DASs is limited in relation to monitoring and/or controlling the physiological and psychological status of the driver. This gap is a major problem that urgently needs to be resolved for the development of AI-based smart cockpits.

Earlier efforts in health monitoring focused on utilizing AI-enabled wearables and biometric data for stroke risk assessment. Multimodal data fusion can further enhance stroke risk prediction [11,18], showing promising applications in improving stroke risk assessment. The smart cockpit is changing from working for the system and physical status of the vehicle to working for the driver and their physiological and psychological states. These systems can identify potential health risks and allow for early intervention through the use of modern sensor technologies, artificial intelligence, and machine learning algorithms [19,20,21]. Diagnosed cardiovascular diseases (CVDs) are some of the leading causes of death globally for about 17.9 million people per year [19]. Technologies such as IoT, mobile health (mHealth), and machine learning have recently become important research directions for the detection, prediction, and monitoring of these diseases [19]. Smart cockpit solutions enhance early detection and response mechanisms for acute health events that would take place while driving with these technologies. In the field of health risk intervention, Ortega-Martorell et al. [18] studied the application potential of AI in the personalized management of atrial fibrillation and stroke, covering the entire process from disease prevention to rehabilitation. Furthermore, AI has great potential in the field of cardiology as it can help doctors diagnose and directly treat patients. Moreover, it can continuously monitor patients to enhance their performance and cut Healthcare System costs [20].

### 1.3. Purpose and Significance of This Review

This review investigates the applications of artificial intelligence in intelligent driving cockpits, particularly focusing on sudden illness detection, health risk intervention, and future development prospects. It also illustrates the technological and infrastructural underpinnings needed to achieve in-cabin health monitoring by systematically studying key technologies such as multimodal biosignal acquisition, edge computing and in-vehicle AI platforms, and in-cabin–out-of-cabin data fusion and management. The review presents a comprehensive study on the application of AI models for the early identification and risk prediction of acute diseases, including arrhythmia, hypoglycemia, and acute respiratory distress; data fusion mechanisms inside and outside the vehicle; and finance and the realization of closed-loop management from monitoring to intervention through vehicle–cloud collaboration. Moreover, the review discusses how AI could enable a smart cockpit to perceive the driver’s health status in real time, provide risk warning, and intervene proactively. Further, it elucidates the ethical, regulatory, and data privacy challenges of such systems. It ends by imagining how smart cockpits will connect with ecosystems such as smart transport and smart healthcare.

This review aims to provide a theoretical foundation and technical reference for the design and development of intelligent driving cockpits, which holds significant theoretical and practical value for enhancing driving safety, improving user experience, and promoting the integrated development of smart transportation and smart healthcare. This work offers a comprehensive and up-to-date reference framework of AI-driven health monitoring technologies in smart cockpits for academic researchers, automotive engineers, product designers, and policymakers. Moreover, it not only aids in understanding of the current state and technical bottlenecks of the field, but also provides theoretical and practical insights for fostering technological innovation, accelerating industrial applications, and establishing robust regulatory and standardization systems.

## 2. Foundations of Perception and Computation in Intelligent Driving Cockpits

### 2.1. Multimodal Biosignal Acquisition Technologies

Common traditional DMSs simply require the detection of observable behavioral signals such as head position, eye opening, and facial expressions [21,22]. Nonetheless, these behavioral cues tend to be identifiable only after major physiological adaptations of the motorist have taken place. Thus, they would be too insensitive to sense small physiological changes or even medical emergencies [23,24]. For example, tiredness and inattention are two of the main causes of accidents. Monitoring physiological parameters helps provide a more objective, real-time measure of the state of the driver. Table 2 lists some mainstream sensors applied to automotive cockpits; using a range of sensors for physiological monitoring enables smart cockpits to better identify fatigue, stress, cognitive load, and even the risk of disease [25]. Multimodal physiological monitoring provides a real-time and objective assessment of a driver’s condition, encompassing electrocardiogram (ECG), electroencephalogram (EEG), electromyogram (EMG), and galvanic skin response (GSR), thus providing a good basis for a comprehensive physiological and psychological understanding of the driver [25]. This approach employs combinations of distinct physical and chemical sensing modalities for a better estimation of human health [26]. For instance, ECG shows the heart condition and stress level [27]; EEG measures brain activity, fatigue, and cognitive status; and EMG checks for muscle activity and fatigue; which is important for detecting fatigue and lapse. Wearable sensor systems of level 4 are hybrid multimodal systems that amalgamate various physical and chemical sensing mechanisms for exhaustive evaluation of the health status of humans [28].

**Figure 2:**
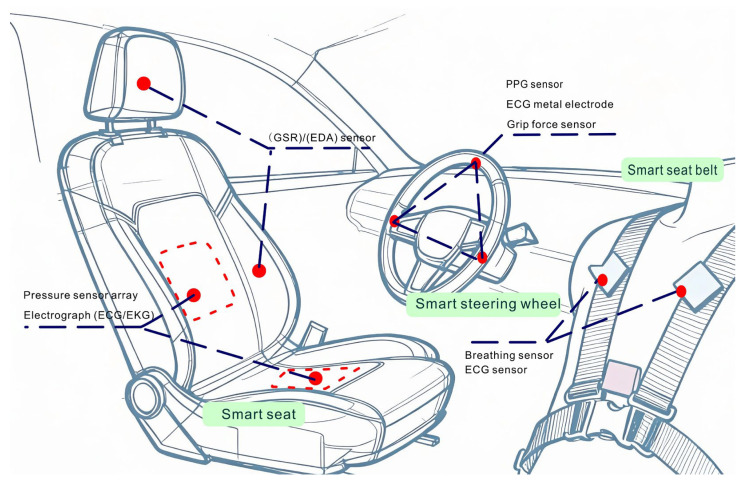
Schematic diagram of contact sensor distribution in the intelligent cockpit of a car.

Contact-based sensors need to be in continuous contact with the human skin to monitor physiological parameters. Wearable physical sensors appeared as early as 1962, and on the backdrop of further technological sophistication, multimodal wearable physical sensors emerged such as the Fitbit Ultra, which measures multiple physical parameters at once [29]. Early wearable sensing tech explorations in 2016 focused on multimodal epidermal chemical sensors, capturing temperature and biochemical signals [29]. As seen in Figure 2, smart seats with pressure sensor arrays and ECG dry electrodes are crucial. They collect physiological data for metrics like HRV. Side-mounted GSR sensors measure skin conductance for emotional arousal assessment. The steering wheel is integrated with PPG and ECG modules. The metal electrode forms a loop, which can capture real-time signals. The grip force sensor can monitor whether the hand force has signs of fatigue.

Besides discrete sensors, smart fabric materials are a promising trend. Integrating sensing components into textiles, they blend sensors into the cabin, balancing function and comfort. Sensors have been developed from textile materials which can read ECG, EMG, body temperature, respiration, pulse, and SpO2, as well as body pressure [30]. Sensor nodes, data acquisition units, and communication modules together form a wireless health monitoring network, enabling real-time and continuous monitoring [31]. In addition, triboelectric nanogenerators (TENGs) represent self-powered, low-cost, and highly customizable sensors that can convert mechanical energy into electrical energy; they are capable of powering wearable electronic devices while simultaneously sensing various biosignals and motion patterns [30]. By adjusting to their environment, TENGs that are implanted in seats or steering wheels can observe a driver’s posture and grip force, facilitating the assessment of their fatigue condition. One of the great significances for intelligent driving cockpit operation over time is the reduced dependence on an external power source and lower maintenance cost of self-powered TENGs.

**Figure 3:**
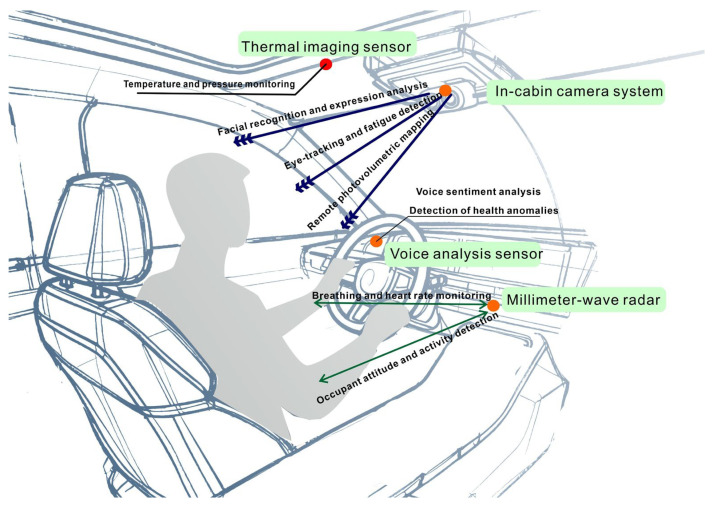
Schematic diagram of non-contact sensor distribution in the intelligent cockpit of automobiles.

Sensors that do not make contact with the body can acquire the physiological signals of a subject without the discomfort of a contact method. Similarly, these sensors can be useful for long periods of time use in confined spaces, such as the driving cockpit where the environment is small. Figure 3 shows that intelligent cameras are capable of reading the facial expression and body language of the driver to determine their emotional state and level of fatigue [32]. The system classified fatigue by analyzing eyelid closure, blink, and yawning frequencies using deep learning. Through analyzing features related to eyelids, the capture and interpretation of micro-expressions and eye movement behaviors used real-time analysis to increase the accuracy of fatigue and emotion recognition [33]. Millimeter-wave radar technology can identify faint micro-movements of a person in order to estimate vital signs such as respiration and heart rate [34]. Smart cockpit assistants powered by AI employ data fusion techniques to assess the driver’s condition. The system could be utilized to warn the car driver or change the vehicle’s settings automatically when signs of fatigue and abnormal heart or respiratory rates are detected [35]. Using a non-contact monitoring approach is both discreet and non-intrusive, assuring health and safety and reliability during monitoring without interference.

Besides physical physiological signals, the driver’s physiological behavior, such as their steering behavior, fatigue level, and attention state, contributes to determining their health status. It is possible to obtain fine-grained perceptions of driver behavior through their driving. For example, sensors in the steering wheel can measure actual driving data with great accuracy, including steering angle, grip strength of the wheel, and micro-corrections made by the driver to maintain a straight line. Such parameters are quite significant since they will reveal characteristics like tension, handling history, or the stress level of the acting individual [36]. Technologies such as facial recognition and eye-tracking can be combined to create smarter fatigue warning systems. The analysis of eye and head movement enables the construction of accurate fatigue models [37]. Utilizing high-sampling-rate EEG and ECG data together with vehicle motion data enables the development of predictive models that can estimate the driver’s cognitive load or emotional state [38]. A strong ability to process and integrate heterogeneous medical image data is shown by deep learning models. In sleep disorder detection, when EEG, EOG, ECG, and EMG signals are fused together, it enhances the accuracy of diagnosis, thus improving health monitoring and enabling earlier intervention [39].

### 2.2. Edge Computing and In-Vehicle AI Platforms

In smart cockpits, data processing and decision making in real time entail extremely demanding latency requirements. By harnessing edge computing, delays in data transmission will be greatly reduced, leading to faster response speed if computational resources are deployed close to the source of data [39,40]. This enables faster processing of the drivers’ biosignals and behavioral data, thus allowing for more timely health risk alerting and intervention [41,42,43]. Consequently, edge computing and in-vehicle AI platforms have become indispensable computing pillars for smart cockpits [44]. Specifically, edge computing enables swift processing of biosignals and behavior information within the cockpit, enabling real-time health-risk detection and adaptive intervention [45]. In one of the studies that address the real-time issues of object recognition, i.e., detecting and localizing the objects in intelligent transportation systems, AI algorithm models were proposed to optimize data loading and offloading strategies in a sensor-based edge environment [46]. This will improve the efficiency of resource allocation and operational performance, as well as ease the load on the cloud, offering better scalability and confidentiality of data [47]. Within the interconnected health realm, Edge Intelligence (EI)—the fusion of artificial intelligence and edge computing—performs computational tasks close to data sources to reduce latency and offload burden from cloud systems [48]. Although processing large amounts of real-time data is feasible through edge computing, the support of cloud computing is still vital for processes that require big data storage, model training, and longer-term analytics [49]. Cloud computing is basically a robust back-end for smart cockpits. As per [50], cloud and edge computing may collaborate in a hierarchical fashion for data processing to meet different application requirements in terms of latency and computational power.

**Figure 4:**
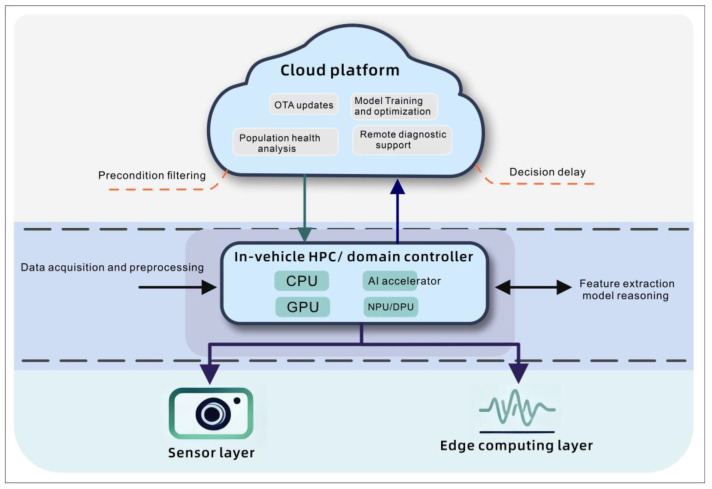
Workflow diagrams of edge computing and in-vehicle AI platforms.

As illustrated in Figure 4, the denoising and filtering processes of sensor data take place before it is fed into the in-vehicle HPC. AI accelerators utilize models like CNNs and LSTMs to extract features and determine the health status of the driver. In the meantime, data privacy at the vehicle level and cloud supports model training, optimization, and over-the-air (OTA) updates. Health-related individual data can be streamed to remote health facilities, in case of emergency, to assist in understanding population-level diseases, hence optimizing personalized models.

The controllers used in them are responsible for the operations of the cockpit devices and for connecting them to the cloud. An autonomous driving domain controller takes substantial data from sensors like LiDAR and cameras and performs perception, decision making, and control in real time [51]. Through the processing of these data, driving patterns can be analyzed, maintenance requirements can be predicted, energy consumption can be optimized, and real-time decision making is made possible for autonomous driving [52]. Advanced driver assistance systems (ADASs), in particular, have artificial intelligence platforms built-in and utilize edge computing systems to identify defects on the road’s surface in order to increase real-time processing capability. Vehicle actuation systems enable precise control of braking, steering, and other operations [53].

In self-driving cars, AI algorithms are used for sensor data, driving behavior, maintenance prediction, energy consumption optimization, and real-time decision making [54]. Table 3 details the functions of various key technologies within the platform’s operation. Smart cockpit sensors and actuators are interlinked and connected through IoT (Internet of Things), representing a network of information exchange in real time [55]. The IoT has far-reaching applications, spanning industries like smart manufacturing, smart homes, and smart healthcare. Wearable sensors, which are a type of IoT device, can collect human physiological data, including heart rate, blood pressure, and activity levels [56]. In addition, these data are then transmitted through IoT to a cloud platform for further processing as well as analysis. In addition, digital twin technology can create a virtual model of the smart cockpit and, by mapping the relevant data in real time, simulate its operating state for predictive maintenance and performance tuning [57]. By using its historical data, as well as AI and ML models that evolve with time, it simulates what vehicles would do in different situations to test the vehicles [58].

In the context of smart cockpits, which handle a substantial volume of sensitive health and driving data, the significance of data security and privacy protection cannot be overstated. A Zero-Trust-based security model has been put forward for 5G industrial Internet collaboration systems. The core tenet of this model is “never trust, always verify”. Under this framework, every access to assets within the industrial Internet undergoes authentication, followed by a comprehensive trust evaluation. As per [59], federated learning and other related methods play a crucial role. These techniques enable local data processing while simultaneously facilitating collaborative model training. By doing so, they effectively safeguard the privacy of users’ data, ensuring that sensitive information remains confidential throughout the data-handling and model-training processes.

### 2.3. Integration and Management of In-Cabin and External Data

The smart cockpit has to be able to interface and manage multisource heterogeneous data from inside and outside the vehicle for comprehensive health monitoring and risk intervention [60], merging internal and external data sources for holistic health assessments and proactive risk management. As shown in Table 4, inside the car, we have the intake of the driver’s physiological signals which may include heart rate, blood pressure, blood oxygen, body temperature, electromyography, etc. These signals can be captured in real time through any in-vehicle camera or through any biometric sensor. This data will be used to assess driver health and detect diseases. Furthermore, computer vision and sensor technologies are used to analyze eye movements, facial expressions, steering operations, voice tone, and other behavioral data. In this way, it attempts to detect fatigue, distraction, or emotional fluctuation and therefore predict driving risks [61].

**Figure 5:**
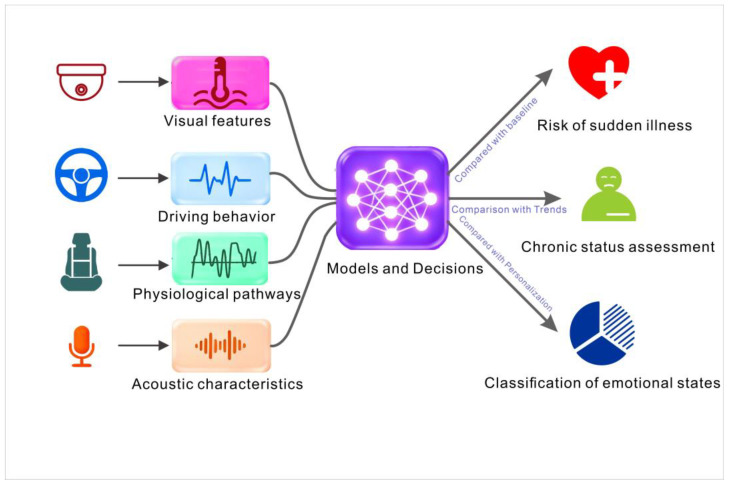
Data fusion and decision making process in intelligent cabins.

The speed, acceleration, and braking patterns detected by vehicle sensors, along with cabin information such as temperature, humidity, and air quality, enable measures of driving comfort to be assessed and adjusted. Artificial intelligence in general, and deep learning in particular, is essential in processing these multimodal datasets to accurately identify the state of the driver and intervene proactively [62]. According to Figure 5, the data layer integrates multimodal visual features, driving behaviors, physiological signals, and acoustic characteristics. The fusion layer uses a combination of attention mechanisms to assign dynamic weights across modalities when learning the model. By comparing individual data with baselines and temporal trends, the system outputs classifications related to acute conditions (e.g., cardiovascular events, epileptic seizures), chronic state assessments (e.g., fatigue levels, stress index), and emotional categories.

External cockpit data primarily focus on information about the surrounding traffic environment, including real-time traffic flow, road conditions, weather, traffic incidents, and V2X communication data. V2X technology enables vehicles to exchange information with infrastructure, other vehicles, and pedestrians—for example, using the IEEE 802.11p standard [63] for vehicle-to-vehicle communication and leveraging advanced networks such as 5G to support the transmission of time-sensitive and safety-critical data [64]. By analyzing these data, AI-assisted systems can generate accident risk maps and provide driving recommendations to optimize driving behavior in potentially hazardous areas, which is essential for comprehensive environmental perception and external risk prediction.

The data fusion and management system is responsible for integrating, cleaning, and preprocessing these multisource, multimodal data, followed by deep analysis using AI algorithms to extract valuable information [65]. For instance, machine learning techniques can fuse multimodal data for early fall prediction, particularly benefiting elderly individuals with neurological disorders. Edge computing platforms can perform preliminary data processing and feature extraction, transmitting valuable information to the cloud for more complex analysis and model training [66]. Blockchain technology, with its distributed storage, peer-to-peer transmission, high confidentiality, and convenient traceability, holds significant potential for ensuring data security and trustworthiness in intelligent transportation systems [67]. Finally, the data fusion and management process must also address data storage, security, and privacy issues to ensure that sensitive health and driving data are properly protected.

## 3. AI-Driven Sudden Illness Monitoring and Early Warning

With the rapid advancement of AI technologies and their widespread application in health monitoring systems, traditional reactive diagnostics are gradually shifting toward proactive prevention and predictive analytics. Table 5 lists the core technologies and application descriptions of AI-driven disease monitoring and early warning. Inside smart cockpits, artificial intelligence merges sophisticated sensing technology, machine learning, and human–machine interaction to offer an unparalleled opportunity for monitoring sudden illnesses and intervening to mitigate health risks to the driver [68]. By using predictive risk models and options for data modeling [69], drivers’ physiology and behavior data can be analyzed in real time to help make an early assessment of health risks. The model can be effective for sudden illness monitoring and early warning.

Machine learning algorithms can learn patterns and make predictions after analyzing large sets of health data. For example, for an individual, the AI models can forecast when diabetes and cardiovascular diseases could occur in the future by analyzing multiple physiologic parameters of patients to issue alerts. Traditional machine learning algorithms including support vector machine, decision tree, random forest, and logistic regression are essential for health risk prediction. Using past medical records, data from wearable devices, and demographic data, algorithms can predict the probability of scenarios such as suffering a stroke, developing diabetes, and having heart disease. Within smart cockpits, these algorithms can identify abnormal patterns that are associated with sudden illness by analyzing the driver’s physiology and behavior [70]. Wearable devices enable models to analyze real-time blood pressure data for the early diagnosis and management of a condition called hypertension. In addition, significant potential for early detection and risk stratification is represented by machine learning for the clinical trial risk assessment of Parkinsons disease, diabetes, and deep vein thrombosis [71]. The effectiveness of these models lies in the benefit they can offer in helping to warn someone before disease onset through learning from complex, high-dimensional data and uncovering subtle patterns. Deep learning, specifically Convolutional Neural Networks (CNNs) and Recurrent Neural Networks (RNNs), can effectively process and integrate complex data from multiple sensors to improve disease prediction [72]. Techniques for multimodal data fusion are utilized to combine data from electronic health records (EHRs), wearable devices, genomics, and medical imaging to enhance predictive accuracy and intervention timing for personalized health and disease management [73]. For instance, to enhance early detection and assess disease progression of chronic kidney disease (CKD), predictive algorithms employing artificial intelligence for healthcare are needed, implementing a wide range of data like genetic profiles, imaging studies, lab results, and clinical records.

Unsupervised learning algorithms are efficient in identifying the patterns and anomalies in a dataset [73]. Effective algorithms may be used to track anomalies in drivers’ behavior and their physiological parameters in smart cockpits. One application of clustering algorithms is for a driver, where the normal data that is used is the driver’s physiological data such as heart rate variability (HRV) and respiration rate (RR). The algorithm then determines other data that is different from this normal data to provide a warning before an illness develops. Anomaly detection has been used successfully in areas such as engine health monitoring, where it applies deep learning frameworks to classify health status and detect anomalies [74].

**Table 5:** AI-driven disease monitoring core technologies.

Technical Category	Key Technologies	Application Description	Refs.
Sensing and data acquisition	Contact/non-contact sensors	Real-time monitoring of multimodal signals (e.g., driver facial expressions, voice tone, heart rate variability, blood pressure, respiratory rate)	[67]
Traditional machine learning models	Support Vector Machines (SVM), Decision Trees, Random Forests, Logistic Regression	Predicting risks of cardiovascular diseases, diabetes, strokes, etc., based on historical medical records, demographic information, and real-time physiological features	[70]
Deep learning models	Convolutional Neural Networks (CNNs) (for image/video feature extraction); Recurrent Neural Networks (RNN/LSTM) (for processing time-series physiological signals)	End-to-end prediction of abnormal blood pressure, arrhythmia, sleep apnea	[72]
Multimodal data fusion	Cross-modal feature alignment, dynamic weight allocation, fusion of visual, voice, radar, and wearable physiological data	Improving accuracy and response speed of sudden illness early warning	[73]
Anomaly detection and clustering	Unsupervised clustering (based on Isolation Forest, Spectral Clustering, HDBSCAN); 3D signal density-based anomaly detection	Identifying abnormal driving behaviors (sudden braking, rapid acceleration, direction deviation) or sudden changes in physiological signals to trigger immediate alerts	[73]
Emotion and cognitive load assessment	Dual-branch deep networks, emotion computing models	Real-time monitoring of emotional states (e.g., fatigue, anxiety, anger); automatically adjust cabin lighting/air conditioning or issue voice reminders when emotions deteriorate	[75]
Personalized health baseline	Constructing personal health baselines based on historical EHR, genetic information, and long-term wearable data	Enabling early warning and supporting personalized intervention plans	[76,77]

A driver’s mind and emotions matter a lot; they affect driving and unexpected illness risk. Combining facial expressions, voice tonality, physiological signals, and driving behavior data through multimodal data fusion techniques can provide real-time information regarding driver emotion and cognitive load. A driver emotion recognition system using a dual-branch deep learning network has been employed to continuously monitor the driver’s state for driving safety. Affective analysis, a key part of human–machine interaction, may rely on a variety of data sources—voice, text, images, and physiological signals—to gauge emotions and improve driver safety [75].

In order to facilitate exact health measuring, smart cockpits must establish a personalized health baseline for each driver. Health data collected over the long term should include multimodal characteristics such as static health information (genotype, medical history) and dynamic health information (real-time physiological parameters). AI models can compare real-time data with the personalized baseline through advanced algorithms to catch minor divergences that may be the starting point for sudden diseases [76]. For instance, personal health profiles based on deep learning can be utilized for early detection and monitoring disease progression of CKD [77]. In general, AI-based early warning systems combine and blend multimodal data to effectively provide eligible users and patients with real-time and individualized monitoring of health status, which provides alerts ahead of sudden illness and allows for more accurate interventions.

## 4. Intelligent Health Risk Intervention Strategies in Smart Cockpits

### 4.1. AI-Driven Personalized Interventions

According to research, smart cockpits can alert drivers of their health status and more via voice prompts, haptic feedback (i.e., seat vibrations), or visual alerts [78]. An example is as follows: if the system detects that the driver is becoming fatigued or inattentive, it can recommend taking a break or adjusting the driver’s position. Real-time changes to this adaptive feedback can be made based on the driver’s responses and modifications to the driving environment. The smart cockpit can automatically adjust the environment according to the state of the driver’s body and mind such as temperature, lighting, music, and smell, to help them stay awake [68]. When the system detects increased stress levels, an automatic response can turn on soothing music and dim the cabin lights. These contextual adaptable changes in the environment relieve stress for drivers [68]. By comprehensively assessing the driver’s health status and the current driving context, AI systems can provide personalized health recommendations [79]. For example, during long-distance driving, the system can suggest appropriate rest times and locations based on the driver’s fatigue level and travel schedule. For drivers with specific health risks, the system can even offer recommendations related to disease management, such as reminders to take medication on time or perform brief relaxation exercises.

**Figure 6:**
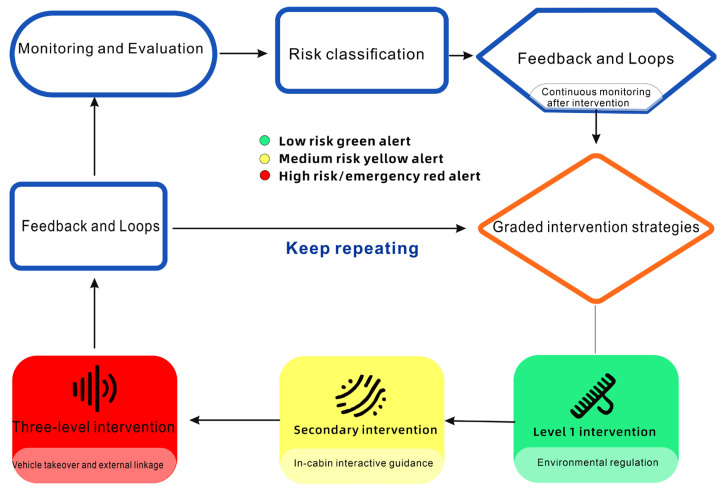
Flowchart of disease risk grouping and intervention.

Once the AI system identifies potential health risks or signs of sudden illness, the smart cockpit initiates a series of intervention strategies aimed at mitigating risk and ensuring driver safety [78]. Tailored interventions are provided based on the driver’s individual characteristics and real-time health status. As illustrated in Figure 6, the monitoring layer receives output on the multidimensional health indicators by the AI models, while the decision layer categorizes risk into three levels:

Low risk (green): Triggers environmental adjustments in the cabin, such as cool-toned ambient lighting, mint scent, and seat vibrations.

Moderate risk (yellow): Initiates proactive guidance, including voice suggestions to rest, navigation to service areas, or reminders to postpone meetings.

High risk (red): Executes emergency responses, such as automatic safe parking, emergency calls, and remote transmission of medical data.

After interventions are implemented, their effectiveness is continuously monitored. For example, if fatigue is not relieved after a level-one intervention, the system automatically escalates to level two. Drivers can manually disable non-emergency interventions, and all operations are logged locally to support continuous AI model optimization, forming an adaptive “assessment–intervention–feedback” closed loop.

### 4.2. Intelligent Emergency Response Mechanisms

In emergency situations such as stroke or cardiac arrest, the smart cockpit must rapidly activate multi-level response mechanisms. When the system detects that the driver has lost the ability to operate the vehicle, the Advanced Driver Assistance System (ADAS) can immediately intervene. High-level ADASs are capable of gradually taking over vehicle control, including deceleration, lane changes, and safe parking, thereby preventing accidents. Such human–machine collaborative driving is crucial for ensuring the safety of passengers and other road users [80]. The AI system can automatically send distress signals to emergency services, providing the driver’s precise location and preliminary health assessment data. Simultaneously, the system can connect to remote medical platforms, allowing physicians to assess the driver’s condition in real time via in-vehicle cameras and microphones and provide remote guidance until professional medical teams arrive [81]. This remote support is especially critical for solo drivers or emergencies occurring in remote areas.

### 4.3. Synergy with External Ecosystems

Smart cockpit health management capability does not operate in a silo, and requires close collaboration with external health ecospheres, data sharing, and functional integration with various stakeholders such as hospitals, emergency centers, insurance providers, family members, and other smart devices like home automation [81]. Data on the health of drivers can be sent to their personal health records for use by the hospital and the physician during normal diagnostics. In addition, combining with public health monitoring systems, e.g., epidemic early warning systems, the smart cockpit could inform drivers of health risks in certain locations, such as regions where infectious diseases are spreading [82]. Future studies must make efforts to study and describe the explainability of AI models, bias detection, and generalization in driving [83]. With continuous technological innovation and cross-domain collaboration, we will gradually promote the use of AI-driven smart cockpits as powerful platforms to safeguard driver health and safety and realize a more intelligent, secure, and personalized mobility experience.

## 5. Challenges and Future Directions

Though smart cockpits adopting AI technology have great potential in marking unexpected illnesses and acting on health risks, their developments are still faced with several challenges alongside several opportunities for future research (Table 6).

### 5.1. Technical Challenges

Smart cockpits employ sensors to collect data like heart rate, respiration rate, and facial expression. However, these sensors have issues in performance and reliability in complex and dynamic driving situations. An example of the above is photoplethysmography (PPG), which is susceptible to motion artifacts and ambient light variations, causing an unstable heart rate. In addition, there is a need to improve the performance of non-contact sensors (radar, thermal) in more challenging weather or lighting conditions to ensure a continuous data stream for the early warning of diseases.

Deep learning models have demonstrated strong performance in emotion recognition and health risk prediction, but their “black box” nature makes the decision making process difficult to interpret, particularly in the healthcare domain. For example, if an AI model predicts a driver’s risk of sudden cardiac events without clearly explaining the rationale behind its judgment, it may be challenging to gain the trust of both physicians and drivers. In addition, the generalization capability of AI models across different driver populations (e.g., varying in age, gender, or health status) and diverse driving scenarios needs further improvement to accommodate real-world variability [84].

Disease monitoring and risk intervention in smart cockpits demand extremely high real-time performance. Smart cockpits integrate multimodal data—including physiological sensors, driving behavior, and environmental information—to comprehensively assess the driver’s state. However, the heterogeneity among these modalities—such as differences in data types, sampling frequencies, and noise levels—poses a significant challenge in effectively extracting, aligning, and fusing features to fully utilize the information from each modality while avoiding redundancy and conflicts [84]. From data acquisition to AI model processing and decision feedback, the entire pipeline must operate within millisecond-level latency to respond to emergencies, such as sudden loss of consciousness or epileptic seizures. Current developments in edge computing and in-vehicle high-performance computing platforms offer potential solutions, yet achieving real-time execution of complex AI models under limited computational resources while maintaining high accuracy remains a formidable technical challenge.

### 5.2. Ethical, Privacy, and Legal Challenges

Balancing the demand for personalized services with the protection of user privacy is an unavoidable ethical issue in this field. Smart cockpits need to collect large amounts of personal health data, biometric data, and behavioral data from drivers [85]. Conformity with applicable data privacy regulations (e.g., GDPR, HIPAA) along with high-level encryption and security measures must ensure the storage, transmission, and processing of such sensitive data [85].

In monitoring sudden illness, false-positive signals generated by the AI model can incite panic and action while false-negative signals can cause a delay in treatment that can be serious [86]. Therefore, false-positive and false-negative rates must be minimized and the system’s reliability must be ensured through rigorous testing and validation [86]. Building trust between humans and AI requires AI systems that demonstrate high reliability, transparent interpretability, and user-friendly interaction. For example, adaptive feedback and alerts can be used to balance the effectiveness of interventions with the driver’s autonomy [84].

When an AI system intervenes or makes a decision in an emergency and an accident occurs, how should liability be determined? Does it lie with the driver, the vehicle manufacturer, the AI system developer, or the sensor supplier? Current legal frameworks have not yet established clear regulations regarding liability for such emerging technologies, which hinders the widespread adoption of smart cockpit technologies [86].

### 5.3. Future Research Opportunities

In the future, digital twin technology may enable smart cockpits to create a “cognitive digital twin” of the driver, integrating physiological, psychological, behavioral, and health history data to construct a real-time, dynamic virtual replica. This digital twin can simulate the driver’s physiological and cognitive responses under different scenarios, allowing for more accurate prediction of health risks, optimization of personalized intervention strategies, and the provision of deeper-level health management services [86].

With the widespread adoption of autonomous driving technologies, particularly at Level 3 and above, the disease monitoring and health intervention systems in smart cockpits are expected to achieve deeper integration with autonomous driving systems. For example, when a driver is in a suboptimal health state or experiences a sudden illness, the system can seamlessly switch from the manual to autonomous driving mode or coordinate the autonomous driving system to execute an emergency stop. This deep integration will significantly enhance driving safety and provide more comprehensive health protection for drivers [86].

Future research will focus on developing more advanced, comfortable, and unobtrusive biomedical sensors and non-contact monitoring technologies. For instance, non-contact techniques such as terahertz waves and millimeter-wave radar can more accurately measure physiological indicators—including cardiopulmonary function, body temperature, and micro-expressions—without interfering with the driver. At the same time, integrating novel materials and flexible electronics to develop invisible sensors embedded in seats, steering wheels, and other cabin components will further enhance user experience and facilitate convenient data acquisition.

To achieve interconnectivity of health data within smart cockpits, it is necessary to promote the standardization of data interfaces, communication protocols, and data formats. This will help different brands of automobiles, health devices, and external healthcare ecosystems in sharing data effortlessly. Establishing unified evaluation standards and certification mechanisms is also crucial to ensure the safety and effectiveness of AI-driven health monitoring systems [85].

The health management capabilities of smart cockpits will no longer be confined to the vehicle interior but will achieve deep integration with smart city infrastructures and remote healthcare systems [85]. For example, when a driver experiences an emergency health condition, the system can not only automatically call for emergency services but also transmit the driver’s real-time health data to nearby hospitals, thereby saving valuable time for subsequent treatment. In addition, by integrating with public health data, the smart cockpit can provide drivers with personalized health risk warnings and travel recommendations, such as avoiding areas with high infectious disease risk.

## 6. Conclusions

Artificial intelligence has become the core driving force for enabling sudden illness monitoring and health risk intervention in smart cockpits. By integrating advanced sensing technologies and multimodal data fusion, AI can perceive drivers’ physiological (e.g., heart rate, respiration), cognitive, and emotional states in real time, effectively identifying fatigue, distraction, and even early signs of potential cardiovascular or neurological disorders. Machine learning and deep learning risk prediction models have the ability to learn complex patterns from large datasets, achieving high precision in health risk prediction and providing the basis for early warning. In addition, personalized intervention strategies driven by AI, such as adaptive feedback, intelligent cabin environment adjustments, and contextual health recommendations, can provide personalized support to the driver according to individual needs and real-time state, thus reducing the health risk significantly. In times of emergencies, the AI system can cooperate with ADASs to assume control of the automobile and can make an emergency call as well as call remote medical assistance.

Sudden illness monitoring and the health risk intervention of smart cockpits based on AI will be very important for driving safety as well as for the health and safety of the driver. By early warning and timely intervention, this technology can help in reducing the number of car accidents due to health emergency situations, thus protecting the lives and property of drivers, passengers, and other road users. Transitioning driver health management from passive treatment to proactive prevention will improve the overall health of the driver. In addition, it will enhance the quality of life and travel experience with better safety and comfort levels.

Future smart cockpits will hold great promise for health management. Advancements in technology like new biomedical sensors, non-contact monitoring technologies, and cognitive digital twins will further improve the accuracy of the monitoring and intelligence of the intervention. In the future, it may be possible to fully realize the potential of smart sensors, yet this can only be achieved by surmounting current challenges such as sensor accuracy, AI model interpretability, and real-time processing. Furthermore, to drive the future progress of AI applications, close interdisciplinary collaboration among computer science, medicine, psychology, ethics, and law is essential, as it is necessary to address the ethical, privacy, and legal issues encompassing data security, liability allocation, and user trust. With continuous technological advances, robust frameworks, and deep understanding of user needs, AI-based healthcare management systems will mature. With the use of intelligent health management systems, travel in the cockpit will be safer, smarter, and more personalized.

## Figures and Tables

**Table 1:** Typical sudden illness and accident types.

Sudden Illness	Symptoms	Resulting Accident Types
Cardiovascular diseases (myocardial infarction, arrhythmia, heart failure)	Sudden chest pain, loss of consciousness, sudden cardiac arrest	Loss of braking/steering control, leading to straight-line collisions, rollovers, or rear-end collisions
Cerebrovascular diseases (cerebral infarction, cerebral hemorrhage, subarachnoid hemorrhage)	Sudden dizziness, hemiplegia, confusion	Vehicle drift out of lane, collisions with roadside facilities
Large aneurysm/aortic dissection	Sudden severe pain, loss of consciousness, sharp drop in blood pressure	Loss of control collision, missed braking opportunity
Diabetic acute complications (hypoglycemia, hyperglycemic crisis)	Sudden confusion, coma, visual impairment	Uncontrolled collisions, missed braking opportunity
Acute respiratory attacks (asthma, acute exacerbation of COPD)	Dyspnea, hypoxic unconsciousness	Loss of control and lane drift, collisions with roadside obstacles
Acute digestive system diseases (gastric ulcer perforation, acute abdominal pain)	Sudden severe pain, loss of attention	Loss of control of steering, sudden braking leading to rear-end collisions
Sleep apnea syndrome	Drowsiness, momentary loss of consciousness (microsleep)	Fatigue driving leading to rear-end collisions, rollover
Others (sudden pain, syncope)	Sudden dizziness, blurred vision, loss of balance	Multiple types (loss of control, collisions)

**Table 2:** Comparative analysis and suitability evaluation of health monitoring sensors in smart cockpits.

Sensors	Signal Types	Suitability	Technical Advantages	Limitations
Seat pressure sensor array	Pressure distribution, contact area changes	Medium	Captures body posture changes in real time; supports long-term monitoring	Affected by seat material and sitting posture (signal vulnerable to vibration interference)
Seat ECG dry electrode	Heart rate variability data	Medium	High signal stability; enables continuous cardiac activity monitoring; assesses stress levels	Requires continuous contact; clothing obstruction reduces signal quality
Seat Side GSR Sensor	Skin conductance changes	Low	Fast response for emotional state assessment	Susceptible to environmental temperature and humidity; individual skin condition differences may cause data deviation
Steering wheel PPG + ECG module	Optical pulse signal, ECG signal	High	Natural usage, non-invasive	Requires continuous grip; signal loss when hands are off the wheel
Steering wheel grip force sensor	Grip strength changes, pressure distribution	High	High sensitivity; triggers fatigue warnings rapidly	Greatly affected by driving habits; potential sensor wear with long-term use
Smart textile sensor	Pressure, body temperature, ECG, EMG, respiration, pulse, etc.	High	High comfort, supports multimodal signal synchronous acquisition	Performance degradation after long-term washing; maintenance requires overall fabric replacement; currently high cost
Camera (RGB/NIR)	Facial expressions, eye movements, blinking, yawning and behavioral features	High	High comfort; supports synchronous acquisition of multimodal signals	Heavily affected by lighting; easily occluded; privacy concerns exist
Camera (PPG)	Pulse extraction through facial color changes	Low	Completely non-contact, can be analyzed synchronously with expressions	Extremely sensitive to motion and lighting changes, low accuracy
Millimeter-wave radar	Remote acquisition of respiratory, heart rate	Medium	Unaffected by lighting; can penetrate clothing	Susceptible to interference, complex algorithms, accuracy needs improvement
Infrared thermal imaging	Monitoring skin temperature, respiratory heat flux	Low	Works in complete darkness	Relatively high cost, limited resolution

**Table 3:** Key technologies of edge computing and in-vehicle AI platforms.

Technical Module	Function Description	Refs.
Edge computing	Deploys computing resources on the in-vehicle or base station side, reducing network latency to the millisecond level and enhancing real-time response capabilities	[39,40]
In-vehicle AI accelerator	Enabling high-speed feature extraction and health status assessment	[44]
Real-time physiological signal warning	Performs denoising, filtering, and inference on drivers’ biological signals (e.g., heart rate, posture), supporting real-time physiological analysis and millisecond-level health risk alert triggering	[45]
Task scheduling and model offloading	Dynamically determining execution on end, edge, or cloud nodes based on computational complexity and latency requirements to optimize resource utilization	[46,47]
Cloud–edge collaboration	Cloud nodes handle large-scale data storage and model training; edge nodes take charge of real-time inference and desensitized data reporting	[50,51]
IoT interconnection	Implementing unified protocols to enable interconnection of in-vehicle and external sensors and actuators	[55]
Digital twin	Constructs virtual cabin models to map sensor data in real time, supporting predictive maintenance and system optimization	[57]
Zero-trust security model	Implementing identity authentication and trust evaluation for each access to ensure in-vehicle network and data security	[59]

**Table 4:** In-cabin and external data fusion and management.

Source	Category	Data	Application Description
In-cabin	Driver physiological signals	Heart rate, blood pressure, blood oxygen, body temperature, electromyographic signals	Assess health status, identify cardiovascular events, epilepsy and other sudden illness risks
	Driver behavior data	Eye movement trajectory, facial expression features, steering operation frequency, voice intonation changes	Determine fatigue level, distraction status and emotion classification, predict driving risks
	Vehicle dynamic data	Real-time speed, acceleration curve, brake pedal stroke, steering angle	Optimize driving comfort with environmental data, implement active intervention
Cabin exterior	Cabin environment data	Temperature, humidity, PM2.5 concentration, CO_2_ content	Adjust air conditioning, seat ventilation and other comfort configurations
	Traffic environment information	Real-time traffic flow, road curvature, friction coefficient, weather warnings, accident black spots	Achieve environmental perception, path planning and risk warning
Integration and management platform	V2X communication data	Inter-vehicle distance, traffic light status, pedestrian crossing warnings	Adjust speed/braking strategies; optimize comfort control
	Multimodal features	Edge computing for data cleaning, preprocessing and feature extraction	Serve as input for deep learning models; support hybrid attention weight distribution
	Storage/Security layer	Blockchain ledger, encrypted storage	Data integrity, privacy protection, traceability
	Cloud analysis	Large model training, long-term trend learning	Disease detection and chronic condition assessment

**Table 6:** Overview of Key Challenges and Opportunities.

Technical Module	Functional Description
Technical challenges	Camera sensors: Prone to interference from motion artifacts, lighting conditions and other factors, leading to insufficient detection accuracy.
	Non-contact radar/infrared thermography: Performance is limited under harsh weather conditions.
	Deep learning models: The “black box” nature causes poor interpretability and low user trust.
	Model generalization: Insufficient adaptability across different populations and scenarios.
	Multimodal data fusion: Heterogeneity issues lead to difficulties in feature alignment/fusion, paired with limited real-time performance.
	Edge computing resources: Constrained hardware makes it hard to run high-precision AI models within millisecond-level latency.
Ethical, privacy, and legal challenges	Data compliance: A large volume of health and biometric data must meet regional regulations (e.g., GDPR, HIPAA).
	Alert accuracy: High risk of false positives/negatives, requiring error rate reduction.
	Human–machine interaction: Need for transparent, interpretable interaction methods to build user trust.
	Liability division: Unclear accountability when emergency intervention causes accidents.
Future research opportunities	Driver cognitive digital twin: Construct a “cognitive digital twin” for drivers to enable precise risk prediction.
	Integration with autonomous driving: Deeply integrate with L3 autonomous driving to trigger automatic switching or emergency parking when driver health abnormalities occur.
	Non-contact sensor deployment: Integrate new non-contact sensors into seats, steering wheels, etc.
	Data standardization: Promote unified data interfaces, protocols and formats to realize cross-brand/cross-platform interconnection.
	Smart healthcare collaboration: Partner with smart healthcare providers to share transit health data in real time and offer personalized travel recommendations.

## Data Availability

No new data were created or analyzed in this study. Data sharing is not applicable to this article.

## Abbreviations

The following abbreviations are used in this manuscript:

AI	Artificial Intelligence
ADAS	Advanced Driver Assistance Systems
CKD	Chronic Kidney Disease
CVDs	Cardiovascular Diseases
DASs	Driver Assistance Systems
ECG	Electrocardiography
EEG	Electroencephalography
EHRs	Electronic Health Records
EI	Emotional Intelligence
EMG	Electromyography
GDPR	General Data Protection Regulation
GSR	Galvanic Skin Response
HCI	Human–Computer Interaction
HIPAA	Health Insurance Portability and Accountability Act
HMI	Human–Machine Interface
HPC	High-Performance Computing
HRV	Heart Rate Variability
IoT	Internet of Things
LiDAR	Light Detection and Ranging
LLMs	Large Language Models
mHealth	Mobile Health
NLP	Natural Language Processing
OTA	Over-The-Air
PPG	Photoplethysmography
RR	Respiration Rate
SpO2	Peripheral Oxygen Saturation
TENGs	Triboelectric Nanogenerators
V2X	Vehicle-to-Everything
